# Idiopathic massive left ventricular thrombus in HIV patient

**DOI:** 10.1186/1749-8090-7-65

**Published:** 2012-07-04

**Authors:** Natalie Homer, Luke Sheen, Richard Lee

**Affiliations:** 1 Division of Cardiac Surgery, Bluhm Cardiovascular Institute, Northwestern University, 201 East Huron Street, Suite 11-140, Chicago, IL, 60611, USA

**Keywords:** Left ventricular thrombus, HIV

## Abstract

A 47-year old man with HIV presented with a stroke. Imaging revealed a large mobile left-ventricular thrombus. The mass was resected using a small ventriculotomy with good early postoperative prognosis. Thrombus etiology is likely related to HIV pathology.

## Background

LV thrombi typically occur in the case of impaired LV dysfunction as a result of dilated cardiomyopathy, aneurysm or a myocardial infarction [1]. However a thrombus of this size is uncommon, particularly in a patient under 50 years of age with no known cardiac disorder. Because mobile thrombi have a significantly higher risk of embolism than mural thrombi, surgical excision is often indicated [2].

## Case presentation

A 47-year old man with HIV presented with a stroke. Upon workup, he was found to have a large mobile cardiac mass in his left ventricle.

**Figure 1:**
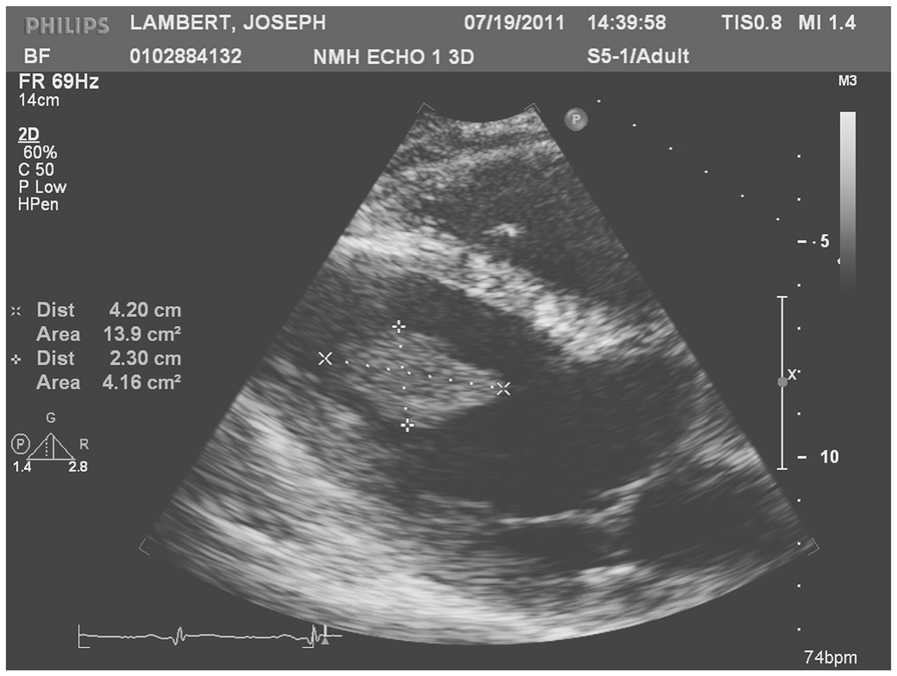
Echo Heart, 2D M-Mode.

**Figure 2:**
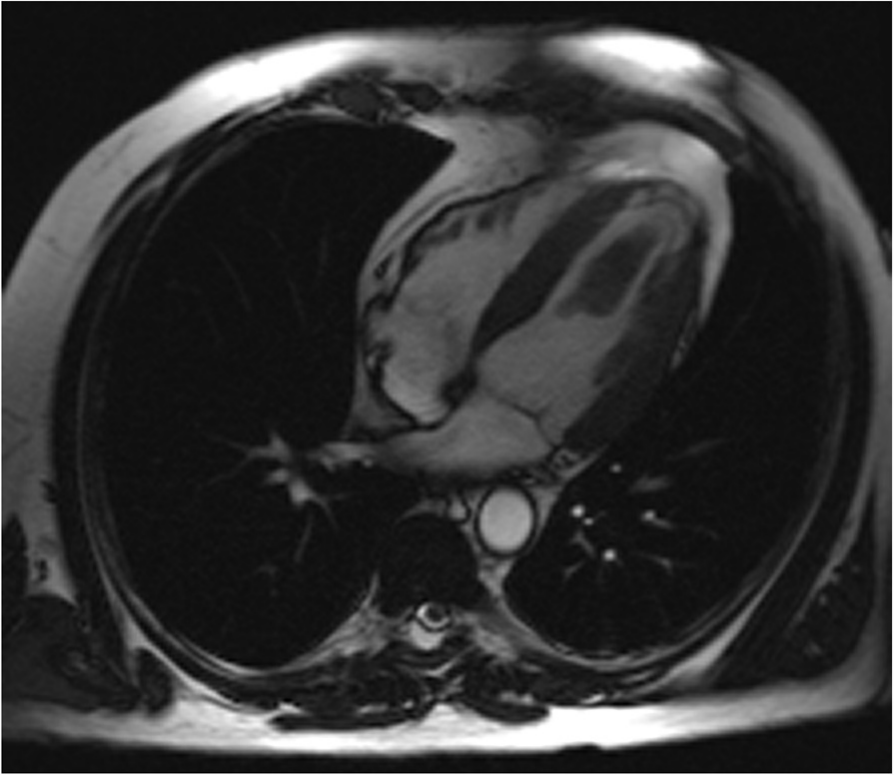
Cardiac MR.

An echo revealed a 5.4 × 2.3 cm smooth, highly mobile surfaced mass to be adherent to the left ventricular apex (Figure 1). The left ventricular size and wall thickness were normal. The apical septum and apex were thin and akinetic. The ejection fraction was 50 %. MR findings confirmed a left ventricular cavitary thrombus adjacent to a region of dyskinetic apex (Figure 2). Associated transmural delayed enhancement with wall thinning of the apex with corresponding wall motion abnormalities. Coronary angiogram was completely normal. Workup was negative for any malignancy.

**Figure 3:**
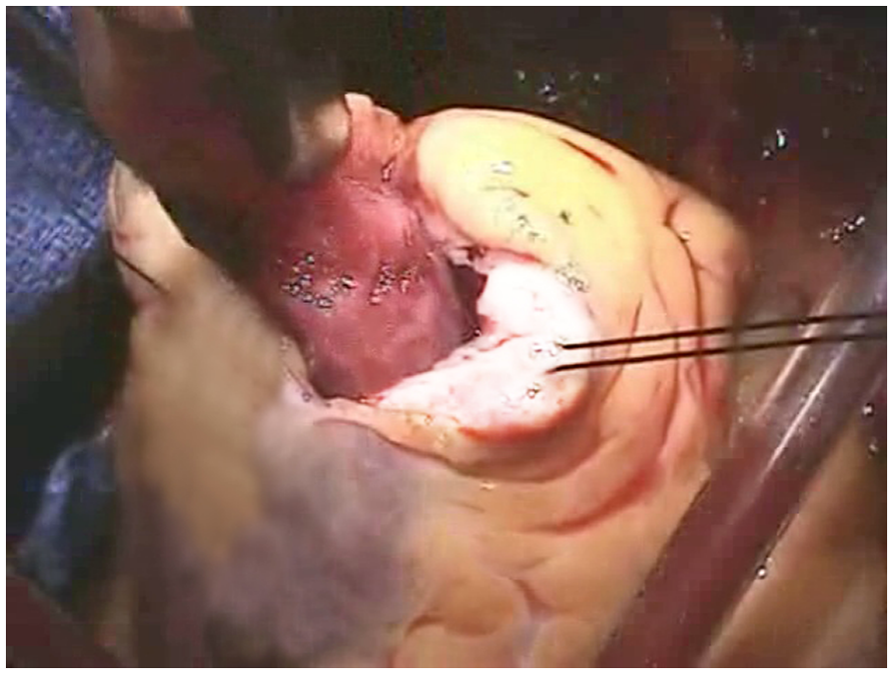
Intra-operative Image of Thrombus Resection.

The mass was removed via ventriculotomy on cardiopulmonary bypass (Figure 3). Pathologic analysis of excised mass tissue revealed fibrin, blood clot, granulation tissue with hemosiderin deposits. This is consistent with an organizing thrombus. Muscle biopsy only showed infarct. The patient experienced complete recovery.

## Conclusions

A thrombus of this size is uncommon, particularly in a patient under 50 years of age with no known cardiac disorder. The patient’s positive HIV status is a possible risk factor for general thromboembolism and microembolism of the coronary circulation. HIV and AIDS are known to be associated with various abnormalities predisposing patients to a hypercoagulable state [3-6]. These pathologies in this HIV positive patient may explain the small myocardial infarct, large thrombus formation and subsequent stroke.

The management of LV thrombi is often restricted to anticoagulant and thrombolytic therapy [6]. However, surgery is indicated in cases with an elevated risk for embolism or for large thrombi where thrombolysis will likely increase risk of embolism [2,7]. This particular patient fit both of those criteria, with a previous stroke and large, mobile thrombus, warranting surgical removal. As the life expectancy in HIV patients continues to improve, this situation may arise again in the future. Other patients may benefit from this approach.

## Consent

Written informed consent was obtained from the patient for publication of this Case report and any accompanying images. A copy of the written consent is available for review by the Editor-in-Chief of this journal.

## Authors' contributions

N.H. complied the images and drafted the manuscript. L.S. provided figures and added to the case presentation. R.L. performed the operation and oversaw the manuscript production process. All authors read and approved the final manuscript.

## Competing interests

The authors declare that they have no competing interests.
